# The Emergency Surgery Frailty Index (EmSFI) in Elderly Patients with Acute Appendicitis: An External Validation of Prognostic Score

**DOI:** 10.1007/s00268-023-06975-w

**Published:** 2023-03-22

**Authors:** Daniele Fusario, Alessandro Neri, Ludovico Carbone, Luca Resca, Luigi Marano, Giulia Gassi, Natale Calomino, Luigi Verre, Franco Roviello, Daniele Marrelli

**Affiliations:** Department of Medicine, Surgery and Neurosciences, Unit of General Surgery and Surgical Oncology, University of Siena, Strada Delle Scotte, 4, 53100 Siena, Italy

## Abstract

**Background:**

Identification of reliable risk-stratification tools is critical for surgical decision making, particularly in frail and elderly. The aim of the study is to validate the Emergency Surgery Frailty Index (EmSFI), in over 65 years old patients operated on for acute appendicitis.

**Methods:**

An observational study was conducted enrolling elderly patients with diagnosis of acute appendicitis who underwent emergency appendicectomy or right colectomy, between 2016 and 2021. All patients were treated according to the last SIFIPAC/WSES/SICG/SIMEU guidelines.

**Results:**

Overall, 61 patients were analyzed. Complication rate was higher for patients in the second EmSFI risk Class. Moreover, ROC analyses identified 3 as the best cutoff value in predicting risk of adverse postoperative events. Complication rate was higher in oldest elderly patients—over 80 years—(42.9 vs 22.5%; *p* 0.05) and was mainly related to medical complications (42.9 vs 12.5%, *p* 0.007). However, intestinal obstruction, peri-appendicular abscess on preoperative CT, peritonitis and a longer duration of surgery are related with increased risk of complications in the group of patients under 80 years.

**Conclusion:**

The EmSFI score results a valid prognostic marker for frailty status, and it may support the surgeon in emergency setting for acute appendicitis. Patients aged 80 years or older have a higher risk of complications, independent from those factors which relate to increased morbidity in younger elderly patients. Age alone is not a reliable indicator of the real surgical risk, but it must encourage the adoption of multidisciplinary collaborative models of care for this group of patients.

**Supplementary Information:**

The online version contains supplementary material available at 10.1007/s00268-023-06975-w.

## Background

The population’s progressive aging and the improvement in the quality-of-life lead to a progressive high prevalence of age-related conditions [1]. It is estimated that about 16% of the global population will be over 65 years by 2050, compared to 9% in 2019 [2, 3]. Elderly patients (≥ 65 years old) represent a significant portion of individuals undergoing abdominal surgery [4]. In these patients, hospitalization for acute event represents a stressful condition related to the increased risk of complications and prolonged length-of-stay [5, 6].

In the emergency setting, provision of effective emergency care is decisive to define prognosis, while, frequently, there is not enough time to complete a full preoperative assessment and to correct any metabolic or systemic imbalance before surgery [7, 8]. A frequent encountered clinical picture is acute abdomen from acute appendicitis, and elderly people often come to surgical observation in advanced stage of disease [9–11]. As the risk of adverse clinical outcomes in older patients is high, the need for identifying reliable risk-stratification tools is crucial for surgical decision making [12].

However, age alone is insufficient to capture the physiologic heterogeneity observed in the elderly, which arises from both comorbidity and variable functional status, merged in the definition of frailty [2, 13–15]. Nonetheless, when surgeons have identified frailty beyond chronological age, they have repeatedly demonstrated that appropriate selection of patients can provide good outcomes [16–20].

In 2021, the ERASO group (Elderly Risk Assessment Surgical Outcome) developed The Emergency Surgery Frailty Index (EmSFI), a multivariable prognostic index based on frail profile and global deficit accumulation [21].

The primary aim of the present study is to validate EmSFI for the estimation of perioperative risk in elderly patients undergoing surgery for acute appendicitis. In addition, we analyzed this recent case series to investigate outcomes and other possible factors which could allow a reliable risk stratification in geriatrics patients.

## Methods

### Study design

This observational study was conducted through the analysis of the data collected in a dedicated database, related to patients with a diagnosis of acute appendicitis undergone to emergency appendicectomy or right colectomy, at the Unit of General Surgery of the University of Siena in Italy. All patients with an age of 65 years or more and treated between 2016 and 2021 were enrolled in the study population.

Clinical decisions were based on the last guidelines for the management of acute appendicitis in elderly patients proposed by the Italian Society of Surgery and Physiopathology (SIFIPAC), along with the Italian Society of Geriatric Surgery (SICG), the World Society of Emergency Surgery (WSES) and the Italian Society of Emergency-Urgency (SIMEU) [22].

The study did not require any modification of the standard therapeutic protocols and was approved by the Ethics Committee of the Area Vasta Sud Est Toscana.

The variables considered were sex, age (“young” elderly subject if 65–79 years old and “oldest” if over-80), associated pathologies, diagnostic data, the EmSFI score, the time between the onset of symptoms and access to emergency ward (EW) and between the hospital admission and the surgery, the type of surgery performed, the 90-days complications and their degree according to the Clavien-Dindo classification [23], the length of hospitalization.

### EmSFI score

The Emergency Surgery Frailty Index (EmSFI) [21] is based on nine variables (Table 1). Four variables are measured in a dichotomous manner, assigning a score of 0 or 1 depending on the absence or presence of the following conditions: age over 80 years, emergency settings, Systemic Inflammatory Response Syndrome (SIRS) state, and history of solid malignancy within the last 5 years. The remaining variables were graded assigning a score from 0 to 2 according to their severity. In particular: “chronic cardiopathy” has score 1 in case of history of cardiac disease or previous percutaneous coronary intervention (PCI) or cardiac surgery, score 2 in case of myocardial infarction within 6 months prior to hospital admission or an acute episode of heart failure within 30 days before the hospitalization; “chronic pneumopathy” has score 1 in case of mild to moderate Chronic Obstructive Pulmonary Disease (COPD), while if severe it has score 2; regarding “other comorbidities” score of 0 is assigned to the modified Charlson Index (mCC) 1–2 [24], a score of 1 to the mCCI 3–5, and a score of 2 to the mCCI   ≥6; concerning “altered autonomy”, a score of 1 is attributed in case of alteration in the performance of daily living activities or cognitive impairment alone, measured with the Italian version of the Barthel Index (IcaBI) [25], whereas a score of 2 to those patients with both conditions; regarding “altered mobility”, a score 1 is assigned to patients showing one criteria between slowness (slow walking speed), shrinking (unintentional weight loss) and exhaustion (self-reported), and a score 2 if more than one of such items.

**Table 1:** Variables for calculating emergency surgery frailty index (EmSFI), proposed by the ERASO group [21]

Variable	Absent	Mild	Severe
Age ≥ 80 years	0		1
Emergency	0		1
SIRS	0		1
Malignancy	0		1
Chronic cardiopathy	0	1	2
Chronic pneumopathy	0	1	2
Other comorbidities	0	1	2
Altered autonomy	0	1	2
Altered mobility	0	1	2

Maximum score: 14 points

Finally, the ERASO group identified three risk class Guided by linear correlation and logistic regression analysis for morbidity and mortality rates; these classes were distinguished as follows:

- First EmSFI risk Class with the maximum score of 3 points,
- Second EmSFI risk Class with total score between 4 and 7 points,
- Third EmSFI risk Class if scored more than 7 points.

### Statistical analysis

The final cohort was stratified in two age subgroups according to chronological age. Clinical characteristics were presented as means with standard deviation (SD) for continuous variable, median with interquartile range (IQR), for categorical variables, or percentages, depending on the variable. The accuracy of EmSFI in predicting surgical complications was analyzed using the area under the curve (AUC). The best point of EmSFI, in terms of sensitivity and specificity, was then reported. The correlation between these variables and the complication rate was studied with univariate analysis by constructing contingency tables and evaluating significance using Pearson's test and with t test for independent variables for the comparison between means (significant value for *p* values <0.05). Statistical tests were conducted with SPSS ver 25.0 program (SPSS statistics, Chicago, Illinois).

## Results

The study population included 61 patients with acute appendicitis (34 males and 27 females), with average age of 76 years (± 8.1). Thirty-nine patients (63.9%) were included in the 65–79 years old group and 22 (36.1%) were older than 80 years. Comorbidities were present in 54 cases (88.5%) and ten patients (16.4%) had at least three comorbidities. Clinical characteristics of the study population are described in Table 2.

**Table 2:** Clinical and surgical data of the study population

	All patients (*N* = 61)	> 80 years old (*N* = 21)	< 80 years old (*N* = 40)
Age	76 ± 8.1	85 ± 4.4	70 ± 4.3
*Sex*			
Male	34 (55.7%)	13 (61.9%)	21 (52.5%)
Female	27 (44.3%)	8 (38.1%)	19 (47.5%)
*Comorbidity*			
Absence	7 (11.5%)	0	7 (17.5%)
1–3	44 (72.1%)	15 (71.4%)	29 (72.5%)
> 3	10 (16.3%)	6 (28.6%)	4 (10%)
*Comorbidities, type*			
Cardiological disease	25 (40.9%)	12 (57.1%)	13 (32.5%)
Respiratory disease	4 (6.5%)	1 (4.8%)	3 (7.5%)
Diabetes	9 (14.7%)	4 (19%)	5 (12.5%)
Neurological disease	6 (9.8%)	1 (4.8%	5 (12.5%)
SIRS	10(16.4%)		
Recent history of solid malignancy	17(27.9%)	7 (33.3%)	10 (25%)
*Frail status*			
Alterated autonomy	2 (3.3%)	2 (9.5%)	0
Alterated mobility	31 (50.8%)	20 (95.2%)	11 (27.5%)
*Laboratory value*			
WBC	13.1/mmc ± 3.4	13.2/mmc ± 3.7	13.1/mmc ± 3.3
Neutrophil	11.7/mmc ± 9.2	14.2/mmc ± 15.1	10.5/mmc ± 2.9
Protein C reactive	13.7 mg/l ± 9.7	15.4 mg/l ± 8.0	12.8 mg/l ± 10.5
Serum creatinine levels	1.07 mg/dl ± 0.4	1.23 mg/dl ± 0.4	1.00 mg/dl ± 0.4
*ASA Score (median)*	2 (1–3)*	3 (2–3)*	2 (1–3)*
*Alvarado score*			
5–6 (Medium diagnostic rate)	8 (13.1%)	1 (4.8%)	7 (17.5)
7–8 (High diagnostic rate)	32 (52.4%)	12 (57.1%)	20 (50%)
9–10 (Certain diagnosis)	21 (34.5%)	8 (38.1%)	13 (32.5%)
*Symptoms*			
Migration of pain	36 (59%)	10 (47.6%)	26 (65%)
Nausea, vomiting	32 (52.5%)	9 (42.9%)	23(57.5%)
Diarrhea	5 (8.2%)	1 (4.8%)	4 (10%)
Intestinal obstruction	17 (27.9%)	6 (28.6%)	11 (27.5)
Tenderness at the right iliac fossa	61 (100%)	21 (100%)	40 (100%)
*EmSFI score*			
1–3 (first class risk)	40 (65.6%)	4 (19%)	36 (90%)
4–7 (second class risk)	21 (34,4%)	17 (81%)	4 (10%)
7–14 (third class risk)	0	0	0
*Delay (hours)*			
Onset symptomatology—EW admission	49.11 (2–240)*	44.8 (9–240)*	51.4 (2–240)*
EW Admission—surgical operation	32.28 (4–140)*	33.5 (6–140)*	31.6 (4–120)*
*Surgical data*			
Timing (minutes)	107.79 (30–305)*	121.2 (50–305)*	107,.8 (30–300)*
Precedent surgery	23 (37.7%)	10 (47.6%)	13 (32.5%)
*Technique*			
Open	20 (32.8%)	8 (38.1%)	12 (30%)
Laparoscopic	29 (47.5%)	9 (42.9%)	20 (50%)
Converted	12 (19.7%)	4 (19%)	8 (20%)
*Type of procedure*			
Appendicectomy	55 (90.2%)	17 (81%)	38 (95%)
Right colectomy	6 (9.8%)	4 (19%)	2 (5%)
*Closure of stump*			
Stapled	26 (42.6%)	9 (42.9%)	17 (42.5%)
Manual	24 (39.3%)	7 (33.3%)	17 (42.5%)
Endoloop	5 (8.6%)	1 (4.8%)	4 (10%)
Absent (right colectomy)	6 (9.8%)	4 (19%)	2 (5%)
*Abdominal drain*			
Yes	59 (96.7%)	21 (100%)	38 (95%)
No	2 (3.3%)	0	2 (5%)
*Peritonitis*			
Yes	42 (68.9%)	14 (66.7%)	28 (70%)
No	19 (31.1%)	7 (33.3%)	12 (30%)
*Histology*			
Acute appendicitis	53 (86.9%)	16 (76.2%)	37 (92.5%)
Adenocarcinoma	5 (8.2%)	3 (14.3%)	2 (5%)
Acute appendicitis associated with neoplasm of peri-appendicular organs	3 (4.9%)	2 (9.5%)	1 (2.5%)
*Complication*			
Absence	43 (70.5%)	12 (57.1%)	31 (77.5%)
Clavien-Dindo I	5 (8.1%)	3 (14.3%)	2 (5%)
Clavien-Dindo II	10 (16.3%)	5 (23.8%)	5 (12.5%)
Clavien-Dindo III	3 (4.9%)	1 (4.8%)	2 (5%)
Clavien-Dindo IV	0	0	0
Clavien-Dindo V	0	0	0
*Median hospital stays (days)*	9 (2–36)*	9 (4–24)*	7 (2–36)*

*SIRS* Systemic Inflammatory Response Syndrome, *WBC* White blood cell, *ASA* American Society of Anesthesiologists, *EmSFI* Emergency Surgery Frailty Index, *EW* Emergency ward. *interquartile range (IQR)

At admission to the EW, mean value for blood tests were leukocytes 13.1/mmc, neutrophils 11.7/mmc, serum creatinine 1.07 mg/dl. Eight patients (13.1%) had an Alvarado score [26] of 5–6, 32 had 7–8, and 21 (34.5%) had 9–10 score; the median ASA score was 2. All 61 patients presented at physical examination pain on palpation in right lower quadrant, 36 migrating pain (59%), 32 nausea or vomiting (52.5%), 17 intestinal obstruction (27.9%) and five diarrheas (8.2%).

Abdominal X-ray was used in 65.6% of cases, abdominal ultrasound in the 83.6% and CT in 64% of cases. As for the preoperative data, the only significant difference between the different age groups was the PCR value, higher in the over 80 group (15.45 vs 12.85 mg/dl, *p* 0.05).

Patients reached the EW with an average delay of 49 h from the onset of symptoms and were brought to the operatory theater with an average time frame of 32 h. The average operative time was 107 min (30–305 min), and the hospital stay was 9 days. The surgery was performed laparoscopically in 29 patients (47.5%), with open surgery in 20 patients (32.8%) and in 12 cases conversion was necessary (19.7%).

In 90.2% of cases an appendectomy was performed, while in six cases (9.8%) it was necessary to perform a right hemicolectomy. The appendicular stump, in cases where only the appendectomy was performed, was closed with a stapler in 29 cases, manually in 26 cases and in five cases by endoloop. A diffuse peritonitis was found in 42 patients (68.9%). At the end of surgery, an abdominal drainage was placed in 59 patients (96.7%).

Pathology reports confirmed an acute appendicitis in 53 patients (86.9%), while an adenocarcinoma of the appendix in five patients (8.2%) and an acute appendicitis associated with a neoplasm of peri-appendicular organs were found in three patients (4.9%).

According to the classification of surgical complications proposed by Clavien-Dindo [23], we observed 5 grade I, 10 grade II and 3 grade III complications in the early 90 postoperative days.

The complication rate was significantly higher in patients over 80 years of age (42.9 vs 22.5%, *p* 0.05). In detail, in this group of patients the higher incidence is related to medical complications, including non-abdominal infectious complications (42.9 vs 12.5%, *p* 0.007) (Table 3).

**Table 3:** Distribution of complications for age

	> 80 years old (*N* = 21)	< 80 years old (*N* = 40)	*p*
Complications	9 (42.5%)	9 (22.5%)	0.05*
Surgical	1 (4.8%)	5 (12.5%)	0.33
Medical	9 (4.9%)	5 (12.5%)	0.007*

*Statistical significant (*p* < 0.05)

At univariate analysis the presence of intestinal obstruction (*p* 0.03), peri-appendicular abscess on preoperative CT (*p* 0.02), the intra-operative finding of peritonitis (*p* 0.05) and the longer duration of surgery (*p* 0.05) are correlated with a significantly increased risk of complications only in the group of patients under 80 years. The incidence of complications in patients over 80 years old is not affected by these variables and ranks among the highest values in each subgroup. Moreover, the presence of an elevated creatinine level at the admission is related to a significant increase in complications in both groups of patients (*p* 0.02).

### Stratification using the EmSFI score

The complication rate was significantly higher in patients in the second EmSFI risk Class compared to first EmSFI risk class (12.5 vs 38.1%, *p* 0.02). Population of the second class included 90% of patients aged over 80 y.o, who represented only 19% of patients in the first class, but this difference did not reach statistical significance. Interestingly, no patients belonged to the third EmSFI risk Class.

**Fig. 1:**
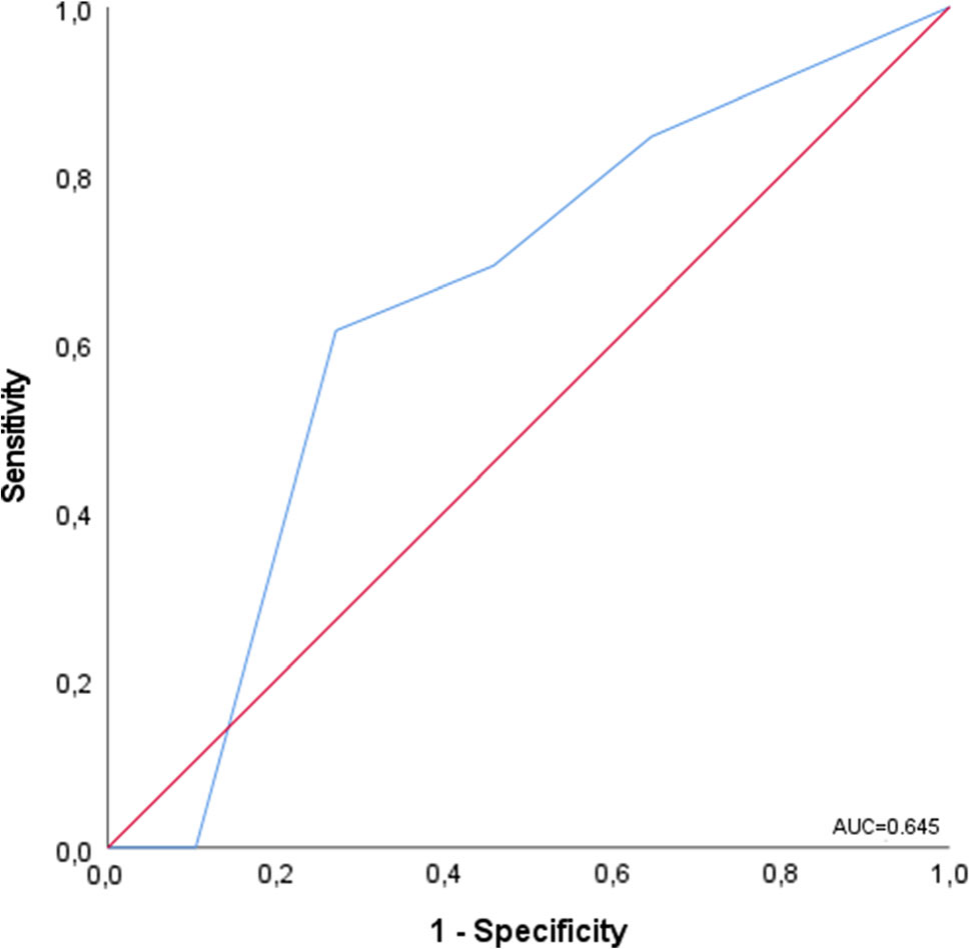
ROC curves (receiver operating curves) for the EmSFI score as predictors of complication

We constructed the ROC curves (receiver operating curves) for the EmSFI score as predictors of complication (Clavien-Dindo ≥ II). The ROC curve identified 3 as the best cutoff value, with a sensitivity of 65.3% and a specificity of 63.5% in predicting the risk of complication, and AUC of 0.645 (Fig. 1).

### Adherence to SIFIPAC/WSES/SICG/SIMEU guidelines

Finally, analyzing our management considering the SIFIPAC / WSES / SICG / SIMEU guidelines, we highlighted how on 17 items (diagnosis, operative treatment, and antibiotic therapy) we respected the recommendations in 100% of cases for 11 items, over 75% for 2 items, between 50 and 75% in 3 items. As regard statement 4.2 we have no data as the whole population analyzed has an Alvarado score >5 (Supplementary Table 1).

## Discussion

With the progressive aging of population, especially in Western countries, several emergency frameworks are now commonly diagnosed in elderly. Acute appendicitis is one of these pictures and in these patients presents a more severe clinical evolution, resulting in a mortality ten times higher than that of adulthood [27]. Indeed, in recent literature, mortality rates of these patients reach 8% and morbidity is reported between 19.3 and 46.2% [22, 28, 29]. Moreover, delays between onset of symptoms and access to the EW and to surgical treatment in the elderly are dramatically dilated [30, 31]. The main reason could be the presence of a nuanced and subtle clinical presentation, which leads to access to hospital and surgical theater only in the already complicated patient. In our series, the analysis of the Alvarado score [26] shows how most of the patients enrolled presented extremely high scores, indicating the presence of “very probable” pathology or acute disease state.

In analyzing the over-65 population, as World Health Organization has defined the "elderly" [32], it was interesting to further distinguish between the “young” elderly (under 80 years old) and the "oldest" elderly (over 80 years old) [33]. In the latter, the higher incidence of comorbidities and the use of more drugs could adversely affect the success of surgery and postoperative outcomes. Nonetheless, these are extremely fragile patients and require particular attention for the significant increase in the mortality rate age-related [34–36].

However, although the population aging correlates with an increase in frailty [37], “older” cannot be synonymous of frailty. Frailty status is comparable to unsuccessful aging, considered reversible, which therefore afflicts a group of individuals with lack of resilience to stressors, such as surgery, and increased risk of high morbidity [38]. Recent literature highlights the importance to add data on performance status, comorbidities, and abilities, to the chronological age in elderly [39]. In other words, the identification of patients at high risk of adverse outcomes is of paramount importance for correct clinical decision and appropriate management [40]. Surgeons need specific and validated surgical risk scores to avoid unnecessary diagnostic procedures and inappropriate medical or surgical treatments in the geriatric population.

In our study, we retrospectively applied the EmSFI to a cohort of patients referred to our high-volume surgical center. Although this is a single-center study, all patients were treated according to the same most recent guidelines for the management of acute appendicitis. The score above 3 points identifies a group of more frail patients, more prone to postoperative complication, and appears a feasible and easy-to-use marker in the emergency setting. Indeed, a recent study [41] applied the EmSFI score to a court of patients with nonmalignant diseases requiring an emergency surgical procedure, adopting a value of at least 4 as a cutoff to define a patient as frail.

In our series, a total of 13.1% acute appendicitis evolved in a contest of appendicular adenocarcinoma or neoplasms of the organs close to the appendix, with the infiltration of neighboring tissues as trigger factor of appendicular inflammation. This data appears perhaps even higher than data reported in other studies (0.5–1%) [42, 43], probably due to the average age of population analyzed and due to the greater number of oncological patients reported in our series being our hospital a referral center for a large range of oncological pathologies.

Overall, in our experience no deaths were recorded, and the complication rate experienced was similar to that reported in the literature [11, 44]. Interestingly, the complication rate is statistically higher in the patient over 80, and how this rate resulted related to medical complications. This may support the hypothesis that the older patient, because of his state of physical and cognitive vulnerability, is “per se” more exposed to postoperative complications.

The univariate analysis finally confirmed that factors influencing complication rate in the young elderly (65–80 years old) as the presence of peritonitis, abscess on CT examination, intestinal obstruction, and surgeon-dependent factors, such as timing of surgery and type of approach, do not affect the complication rate in the great elderly (> 80 years old). These data stress that in this population, the major factor which affect a successful surgical outcome are the patient's own comorbidities and his intrinsic frailty, stimulating the surgeon to carry out a correct and thorough preoperative evaluation even in the context of the emergency.

Among other selected predictive factors, age has been widely investigated as parameter affecting surgical outcomes. It is certainly true that the prevalence of frailty increases with age, as it is seen in 26% of patients aged 80 years or older compared with 7% of adults aged between 65 and 75 years [40, 41]. According to the related literature, we highlighted those octogenarians demonstrated an increased vulnerability for adverse events and medical complications including death. However, recent findings have assessed that older patients with the same chronological age could have divergent outcomes and, therefore, an objective measure of patient’s functional reserves becomes fundamental in predicting postsurgical morbidity and mortality rates [37].

Future research should further develop and confirm these initial findings by validating the score in prospective, multicenter studies, thus ensuring even more widespread application in the identification of the frail patients.

## Conclusion

Acute appendicitis is an important picture of surgical pathology and is becoming an increasingly frequent pathology in the elderly. The EmSFI score is a useful and simple prognostic marker for frailty status in surgical elderly patients. It may support the surgeon in decision for an adequate healthcare plan and preoperative preparation. To the best of our knowledge, this is the first study that aims to validate the EmSFI score.

Furthermore, the comorbidity rate of the "oldest" elderly patients (> 80 years old) appears to affect the early outcomes more than in the younger elderly. The surgical community should encourage the adoption of a multidisciplinary collaborative model of care for frail and elderly.

## Supplementary Information

Below is the link to the electronic supplementary material.

Supplementary file1 (DOCX 15 kb)
