# Supplementary material for: The Emergency Surgery Frailty Index (EmSFI) in Elderly Patients with Acute Appendicitis: An External Validation of Prognostic Score
Source: World J Surg. 2023 Mar 22;47(7):1713–20. doi: 10.1007/s00268-023-06975-w (PMC10229705; doi:10.1007/s00268-023-06975-w)
Supplement: Supplementary file 1 — Supplementary file1 (DOCX 15 kb) [file 268_2023_6975_MOESM1_ESM.docx]

**Supplementary Tab.1: Compliance to statements of the SIFIPAC/WSES/SICG/SIMEU guidelines** [22]

|  | **STATEMENT** | % of compliance |
| --- | --- | --- |
| **Diagnosis** |  |  |
|  | *Statement 1.1* | 100% |
|  | *Statement 1.2* | 100% |
|  | *Statement 2* | 100% |
|  | *Statement 3* | 100% |
|  | *Statement 4.1* | 60,7% |
|  | *Statement 4.2* | N.A. |
|  | *Statement 4.3* | 83,6% |
|  | *Statement 4.4* | 100% |
|  | *Statement 4.5* | 75% |
| **Operative management** |  |  |
|  | *Statement 8* | 67.2% |
|  | *Statement 9* | 100% |
|  | *Statement 10* | 100% |
|  | *Statement 11* | 100% |
| **Antibiotic therapy** |  |  |
|  | *Statement 13* | 100% |
|  | *Statement 14.1* | 50% |
|  | *Statement 14.2* | 100% |
|  | *Statement 15* | 100% |
